# Dietary Intakes of Recipients of Faecal Microbiota Transplantation: An Observational Pilot Study

**DOI:** 10.3390/nu13051487

**Published:** 2021-04-28

**Authors:** Annabel K. Clancy, Christina Lee, Harrison Hamblin, Anoja W. Gunaratne, Antoinette LeBusque, Eleanor J. Beck, Marie V. Dawson, Thomas J. Borody

**Affiliations:** 1 Centre for Digestive Diseases, 1/229 Great North Rd, Five Dock, NSW 2046, Australia; annabel.clancy@cdd.com.au (A.K.C.); harrison.hamblin@cdd.com.au (H.H.); anoja.gunaratne@cdd.com.au (A.W.G.); antoinette.lebusque@cdd.com.au (A.L.); vic.dawson@cdd.com.au (M.V.D.); 2 School of Medicine, Faculty of Science, Medicine and Health, University of Wollongong, Northfields Ave, Wollongong, NSW 2522, Australia; cl685@uowmail.edu.au (C.L.); eleanor@uow.edu.au (E.J.B.)

**Keywords:** faecal microbiota transplantation, dietary intake, fibre

## Abstract

This study reports on the dietary intake of recipients of faecal microbiota transplantation (FMT), comparing this with dietary guidelines, and investigates the relationship between dietary intake and clinical outcomes. Males and females aged ≥ 16 years with irritable bowel syndrome or inflammatory bowel disease undergoing FMT were invited to complete validated symptom and quality of life (QOL) questionnaires and three-day weighed food diaries. Descriptive statistics were calculated for symptom scores, QOL scores, nutrients, and food group servings, and compared to Australian population norms, nutrient reference values, and dietary guidelines. The relationship between dietary intake, symptoms, and QOL was assessed. Participants (*n* = 18) reported baseline symptoms of urgency, abdominal pain, nausea, and bloating and reduced QOL. Of the participants who completed food diaries, 8/14 met the recommended 30 g of fibre when including supplements. Participants met the recommendations for micronutrients and food groups except calcium, fruit, and dairy/dairy alternatives. There was a non-significant trend towards lower symptom severity scores in participants who met the fibre target. The high degree of variability in participant fibre intakes highlights diet as a key variable that has not been previously controlled for in FMT intervention studies. Future studies examining FMT should include dietary analysis of habitual intake of the recipients and donors.

## 1. Introduction

Faecal microbiota transplantation (FMT) is the infusion of homogenised and filtered stool, collected from a healthy, highly-screened donor, into the gastrointestinal tract of an unwell recipient. FMT aims to alter and diversify the gut microbiota in order to treat an underlying disease. FMT has been repeatedly shown to be a highly effective treatment for recurrent *Clostridioides difficile* infection (CDI) [1,2]. FMT has also shown promising results in the treatment of other gastrointestinal conditions, such as irritable bowel syndrome (IBS) and inflammatory bowel diseases (IBDs), including ulcerative colitis (UC) and Crohn’s disease (CD) [3,4]. A 2017 systematic review and meta-analysis assessing the effectiveness and safety of FMT for IBDs found clinical remission as the outcome for 35% of UC and 51% of CD patients [4]. Similarly, in IBS, the overall clinical response rate was 49%, with two studies also demonstrating improvements in the quality of life (QOL) [5]. However, these mixed results at an individual level highlight that improvements in efficacy are still required.

Dietary fibre is a key facilitator of changes in the gut microbiota. Dietary fibre refers to non-digestible carbohydrates with three or more monomeric units, shown to have beneficial physiological effects on human health [6]. The 2011–2012 Australian National Nutrition and Physical Activity Survey (NNPAS) found that over 70% of adults do not reach the recommended fibre intakes [7]. Furthermore, individuals with gastrointestinal conditions, especially IBDs and IBS, have been frequently reported to have poorer nutrient intakes, and are unlikely to meet the recommended guidelines for dietary fibre [8]. These actions are largely shaped by dietary beliefs and behaviours. For example, Casanova et al. 2016 found that 86% of individuals with IBDs avoided some food groups out of fear of worsening IBD flare symptoms [9]. Similarly, in individuals with IBS, Staudacher et al. (2017) found decreased carbohydrate intake and a lower proportion of individuals meeting calcium recommendations on a low FODMAP diet. QOL was also found to be affected by anxiety associated with dining out, economic burdens, or a lack of dietary education [10].

Fibres with fermentable characteristics are substrates for microbial populations in the colon, leading to the production of various metabolites, including short chain fatty acids [11]. Dietary fibre interventions in healthy participants can influence bacterial abundance, especially *Bifidobacterium* and *Lactobacillus* spp. [12]. Dietary fibre may therefore maintain the levels of beneficial bacteria, and thus may support successful FMT treatment. The available literature on the potential role of dietary fibre in supporting FMT, however, is minimal. An abstract study showed a greater likelihood of successful CDI treatment with FMT in individuals with higher fruit and fibre consumption [13]. A study by Wei et al. (2016) compared the outcomes of individuals with UC receiving FMT and a known prebiotic fibre (pectin) supplement compared to FMT alone. Their results suggested that supplementation with pectin delayed the loss of diversity of transplanted gut microbiota and enhanced the effects of FMT [14]. Similarly, a study comparing the outcomes of individuals with constipation receiving a soluble fibre supplement and FMT vs. FMT alone showed significantly improved stool frequency and consistency in the supplement group [15]. Conversely, a South Australian study by Costello et al. reported on the dietary fibre intake of participants at baseline (prior to FMT) below the Australian recommendations of 25–30 g/day for both groups (19 g vs. 21 g for FMT and the control, respectively) [10,11]. There was no relationship between fibre intake and FMT outcomes, and dietary fibre intake post-treatment was not reported in this study [16].

Increasing our understanding of the dietary intakes of individuals undergoing FMT is critical on two accounts. It may inform further research to understand the role of all aspects of diet in FMT, and, most importantly, provide informed clinical recommendations to individuals undergoing FMT. Therefore, the aim of this pilot study was to report on the dietary intake of individuals undergoing FMT and to compare intake with current Australian dietary guidelines. An exploratory objective of this study was to investigate the relationship between dietary intake (more specifically, a high fibre diet of >30 g/day) and clinical outcomes (symptoms and QOL) of recipients of FMT. It was hypothesised that individuals receiving FMT would not be consistently meeting the recommended high fibre target (30 g/day), and secondly, that individuals undergoing FMT and incorporating a consistently high fibre diet will have better clinical outcomes (symptom reduction and QOL).

## 2. Materials and Methods

This study was a prospective pilot study assessing the dietary intake of individuals with various gastrointestinal disorders (IBS, UC, and CD) receiving FMT at our centre. The study was intended to provide information for developing dietary education resources for use in clinical practice and directing future interventional research studies. The study was ceased early due to the COVID-19 pandemic, and, subsequently, the minimum sample size (*n* = 31) was not met [17].

### 2.1. Background to FMT Program at Our Centre

During the study period (September 2019 to March 2020), the FMT program at our centre comprised individualised combination antibiotic pre-treatment. For participants with IBDs, antibiotics and anti-inflammatory medications were used to induce remission, defined as faecal calprotectin < 50 ug/g. Antibiotic pre-treatment was followed by FMT administered in decreasing frequency over a six-month period. The first FMT infusion was administered via colonoscopy, and all subsequent FMT infusions were administered via rectal retention enema at our centre or the participant’s home. Enemas were administered in decreasing frequency from five per week to one per fortnight, and participants received a total of 32 FMT infusions over the six-month period. FMT infusions were prepared according to national and international standards from stool donations from highly screened donors [1,18]. All participants were instructed to follow a high fibre diet (minimum of 30 g/day), and were provided with basic dietary information by their clinical nurse. They were also encouraged to take the prebiotic supplements inulin and pectin. 

### 2.2. Study Procedures

This observational pilot study was approved by the central human research ethics committee. Inclusion criteria were males and females aged ≥16 years of age diagnosed with IBS, UC, or CD who were undergoing the FMT program at our centre. Those who consented to participate were asked to complete validated clinical symptom questionnaires, the abdominal symptoms questionnaire [19], and the SF-36 QOL questionnaire [20] at baseline, week four, week 12, and week 24 of treatment. Participants were also asked to complete a three-day weighed food diary at week four, week 12, and week 24. A baseline food diary was not collected due to participants completing a low residue bowel preparation diet prior to their initial colonoscopy and FMT infusion. Participants were asked to record all foods, beverages, and supplements consumed over a three-day period (including one weekend day). Food diaries were assessed by a trained dietitian, and the dietary information was clarified with the patient if required. Clinical data including demographic data, medical history, colonoscopy reports, and pathology reports were also recorded from the participant’s medical records.

### 2.3. Data Analysis

De-identified data was entered into an Excel database for analysis. Dietary intake data was analysed using dietary analysis software (FoodWorks v.10 Xyris Software Ltd., Brisbane, QLD, Australia) [21], drawing upon the most recent Australian food, supplement, and nutrient database [22]. Goldberg cutoffs were calculated and applied. The mean reported energy intake was deemed to be within acceptable ranges for the population [23]. Statistical analysis was conducted using GraphPad Prism Version 9.0 for Windows (GraphPad Software, San Diego, CA, USA). Descriptive statistics (mean/median, standard deviation, and range) were calculated for symptom scores, QOL scores, intake of nutrients (e.g., fibre), and food group servings. A Kruskal–Wallis H Test was used to assess the differences between indications at baseline. Baseline QOL scores were compared to published Australian population norms [24] using an independent *t*-test. The following assessments were conducted at the week four and week 12 time points: nutrient intakes were compared to Australian nutrient reference values (estimated average requirements (EAR) or adequate intake (AI)) [25]. Fibre intakes were compared to the 30 g/day recommendation provided to all participants. Food group intake was compared to the Australian dietary guidelines [26]. The relationship between dietary intake, symptoms, and QOL were assessed by Pearson’s correlations or equivalent. Changes in symptoms and QOL over time were assessed using the Wilcoxon signed-rank test. Week 24 was not analysed due to the low number of responders. Statistical significance was set at 5%. 

## 3. Results

### 3.1. Baseline

A total of 18 individuals (*n* = 8 males, median age 35 years; seven with IBS and 11 IBDs) consented to participate in the study (participation rate = 62%). Participants reported baseline symptoms of a median of two bowel motions daily, urgency, abdominal pain, nausea, and bloating (Table 1). All aspects of QOL except physical functioning were found to be significantly lower than the Australian population norms (all *p* < 0.001). There were no significant differences in age, gender, or BMI identified between IBS, UC, or patients with Crohn’s disease. QOL component role limitations of physical functioning, bodily pain, and social functioning were significantly lower in patients with IBS.

Participants with IBS (3 IBS–Constipation, 1 IBS–Diarrhoea, 3 IBS–Mixed type) predominantly reported symptoms of epigastric pain, bloating, and postprandial fullness. All participants had received pre-treatment with vancomycin alone (*n* = 3) or with the addition of rifaximin (*n* = 2), tinidazole (*n* = 1), or ciprofloxacin (*n* = 1). One participant also had concomitant coeliac disease. Considering QOL, participants with IBS scored significantly lower for role limitations due to physical functioning, bodily pain, and social functioning than the IBD groups (Table 1). 

Participants with IBDs (4 UC, 7 CD) predominantly reported symptoms of bloating and urgency. The majority of participants with IBDs (*n* = 8/11) were in clinical remission based on faecal calprotectin results (median 20 ug/g (9–145 ug/g)). Participants with CD had received pre-treatment with combination anti-*Mycobacterium paratuberculosis* spp. (anti-MAP) therapy [27]. Participants with UC had received individualised antibiotic combinations (Supplementary Table S1). No specific medical history was noted. 

### 3.2. Week Four Follow-Up

Fourteen participants (six males) completed the week four follow-up food diary and questionnaires. Improvements in bowel habits were noted, with a median of two formed bowel motions daily (range 1–3). Urgency (*n* = 7), abdominal pain (*n* = 7), bloating (*n* = 12), and flatulence (*n* = 12) remained the most common symptoms. There was a trend towards a reduction in the severity of these symptoms, but this was not significant (data not shown). Similarly, participants reported improvements in all QOL domains, which were not statistically significant. However, all aspects of QOL (except physical functioning) remained below Australian population norms (data not shown). 

Of the 14 participants who completed a food diary, six reported following a specific diet or having a dietary restriction, including two dairy-free pescatarians, with one also gluten-free, vegan, lactose-free, egg-free, and soy-free, and on an energy-restricted ketogenic diet.

Eleven participants reported taking at least one dietary supplement, all of which included fibre within the supplement. Six participants were also taking a vitamin/mineral supplement, and three used a protein powder. Two participants had seen a dietitian for additional dietary support since baseline.

Just over half (8/14) of the participants met the target intake of 30 g of fibre when including supplements, with a median intake of 35 g/day (15–90 g/day). Notably, the vegan and pescatarian participants had the highest fibre intakes. Females also had a higher median fibre intake than male participants (42 g/day vs. 26 g/day, respectively), with 6/8 females meeting the 30 g/day compared to 2/6 males. Almost all females (7/8) met the AI of 25 g/day when including supplements. 

Interestingly, when supplements were excluded from the analysis, the median intake (27 g/day (6–86 g/day)) indicated that participants were below the fibre targets, with 6/14 of the participants consuming greater than 30 g/day. Female participants reported higher median fibre intake without supplements than males (35 g/day vs. 23 g/day, respectively), with 4/8 females and 3/6 males meeting the 30 g target. Just over half of the females (5/8) met the AI of 25 g/day when excluding supplements. 

Three participants were not taking a fibre supplement, with one not meeting the fibre target. Of the 11 participants taking fibre supplements, five (45%) did not meet the fibre target (median 19 g/day (15–25 g/day)). Four (36%) participants would have met their recommended fibre intake without supplementation (median 49 g/day (48–90 g/day)). The remaining two participants effectively used fibre supplements to meet the recommended fibre intake. These participants had received dietary advice from a dietitian.

Dietary analysis (including dietary supplements) of median participant intakes revealed adequate median energy and macronutrient intake for all participants, except the participant reporting to follow a calorie-restricted ketogenic diet (Supplementary Table S2). Median participant intake was also generally above the EAR for all micronutrients, except calcium, with 8/14 participants having intakes below the EAR (Table 2). This further increased to 10/14 participants when excluding dietary supplements. Also of note, a participant following a pescatarian, dairy and gluten-free diet did not meet the EAR for vitamin B12.

**Figure 1:**
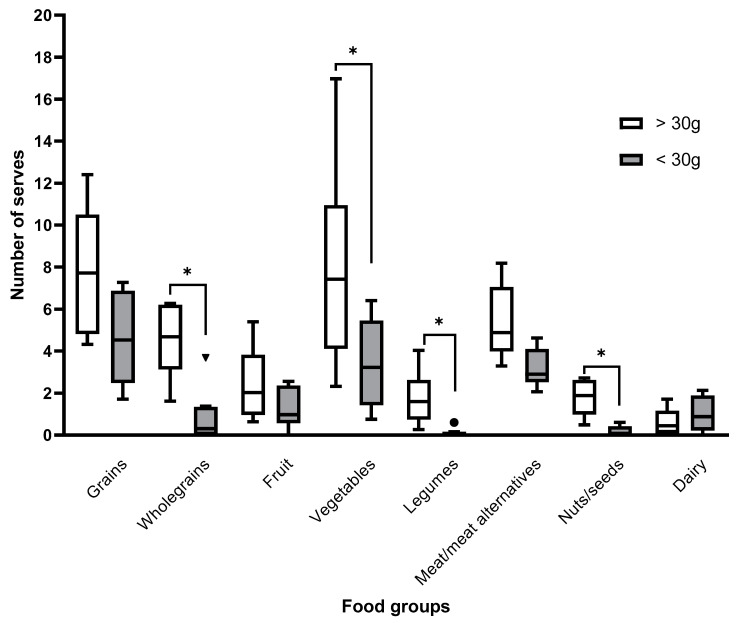
Differences in dietary patterns between participants meeting fibre requirements (*n* = 14). Participants meeting the fibre target of 30 g/day (without dietary supplementation) consumed significantly more whole grains (*p* = 0.002), vegetables (*p* = 0.043), legumes (*p* = 0.001), and nuts/seeds (*p* = 0.002) than participants who did not meet the fibre target. * indicates statistical significance *p* < 0.05. ^●^^▼^ Indicate outliers.

Analysis of dietary intakes by food groups showed median fruit and milk/alternatives intake to be below the recommended guidelines. None of the participants met the recommended servings of milk or milk alternatives. Approximately half of the participants met the recommended servings of fruit (6/14), vegetables (7/14), and grains (9/14). Perhaps unsurprisingly, participants who consumed >30 g of fibre per day (without supplementation) consumed significantly more servings of whole grains, vegetables, legumes, and nuts (Figure 1). 

**Figure 2:**
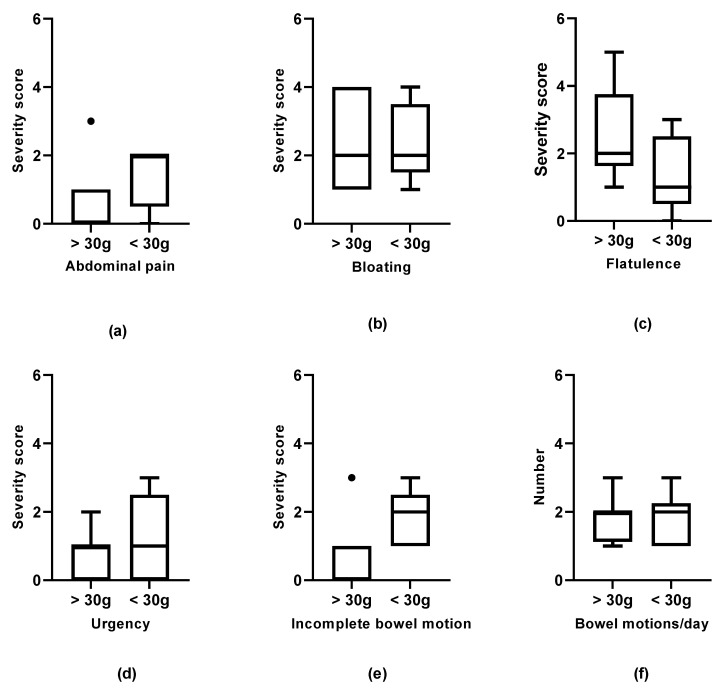
Relationship between the symptom severity score and dietary fibre intake (*n* = 14). Median symptom severity scores were compared between participants who met the fibre target of 30 g/day (*n* = 8) and those with intakes less than 30 g/day (*n* = 6), including supplements. A higher score indicated more severe symptoms. ● Indicates outliers within the data. (**a**) Relationship between abdominal pain and fibre intake. Abdominal pain: 0 vs. 2, *p* = 0.19. (**b**) Relationship between bloating and fibre intake: 2 vs. 2, *p* = 0.88. (**c**) Relationship between flatulence and fibre intake. Flatulence: 2 vs. 1, *p* = 0.16. (**d**) Relationship between urgency and fibre intake. Urgency: 1 vs. 1, *p* = 0.68. (**e**) Relationship between incomplete bowel motions and fibre intake. Incomplete 0 vs. 2, p = 0.08. (**f**) Relationship between bowel motion frequency and fibre intake. Bowel motions: 2 vs. 2, *p* = 0.99.

We examined the relationship between gastrointestinal symptoms, QOL, and dietary intake. Overall, there were no significant relationships identified between meeting fibre requirements and gastrointestinal symptoms or QOL. There was a trend towards participants who met the fibre target having lower symptom severity scores for abdominal pain (*p* = 0.19) and incomplete bowel motions (*p* = 0.08), but higher symptom severity scores for flatulence (*p* = 0.16) (Figure 2). 

### 3.3. Week 12 Follow-Up

A total of six participants (three males) completed the week 12 follow-up. Improvements in bowel habits were maintained (data not shown). Of the six participants who completed a food diary, two reported following a specific diet or having a dietary restriction, including vegetarian, dairy-free, and lactose-free. Three of the six participants reported taking a dietary supplement, with two including a fibre supplement. 

Dietary analysis (including dietary supplements) revealed that participants met the targets for all nutrients, including fibre and calcium, with median intakes of 30 g/day (27–49 g/day) and 995 mg/day (905–1380 mg/day), respectively. However, similarly to week four, when dietary supplements were excluded, the median fibre intake was just below the dietary target (27 g/day (21–54 g/day)). 

Food group analysis showed median vegetable (3.7 (1.8–5.5) servings) and dairy (1.7 (0.9–2.1) servings) intake to be below the recommended guidelines. Similar to week four, none of the participants met the recommended servings of milk/alternatives, a third met the recommended servings of grains and vegetables, and half met the recommended servings of fruit. Due to the small number of food diaries collected at this time point and week 24 (*n* = 2), no further analyses were performed. 

## 4. Discussion

Overall, this pilot study identified that participants presenting with IBS and IBDs undertaking FMT do not consistently meet the recommended intakes of fibre or calcium. Accordingly, they also did not consistently meet the recommended intakes of fruit, vegetables, milk/alternatives, or grains. 

Median participant fibre intake in this study was higher than the reported Australian average of 20.7 g [7] and other studies of individuals with IBDs [8,16,28]. The average intake of vegetables, grain, and meat/meat alternatives were also higher in our study population than the reported Australian average [29] and a Dutch study of individuals with IBDs [28]. Fibre intake reported here was also higher in females than males, consistent with Australian data [7]. Higher fibre, vegetable, grain, and meat/meat alternative consumption may be reflective of dietary advice provided by clinical nurses, a more health-conscious group participating in a dietary study, the participant’s dietary restrictions (pescatarian/vegan), or that the participants here were in clinical remission. Conversely, milk/alternatives intake was below the Australian average [29], with median calcium intake below the recommended guidelines. Low dairy consumption has previously been reported in individuals with IBDs [28,30] due to dietary beliefs of dairy triggering or worsening symptoms. Similar trends for calcium intake have been reported in individuals with IBS [31]. Avoidance of dairy increases the risk of nutritional inadequacy and subsequent conditions, such as osteoporosis, of which many with IBDs are already at increased risk [30]. Self-imposed dietary restrictions are increasingly common, and greater recognition of these are required with the encouragement of consumption of dairy or calcium-rich dairy alternatives considered, especially when recipients are clinically following FMT well.

An exploratory objective of this study was the association between fibre intake and symptoms. While the pilot nature of this project means that significant associations could not be identified, there was a trend towards participants who met the fibre target having lower symptom severity scores for abdominal pain and incomplete bowel motions, but higher symptom severity scores for flatulence. This trend is in line with our hypothesis that a high fibre diet may assist with the maintenance of new microbiota introduced by FMT human studies [13,14,15,32] and subsequently sustaining increases in beneficial short chain fatty acids [33]. Previous work in individuals with IBS suggests that low dose prebiotic supplementation of < 6 g may be associated with improved symptoms, while higher doses or inulin-type fructan supplements may increase gastrointestinal symptoms, such as bloating and flatulence [34]. Subsequently, a U-shaped relationship between fibre and symptoms may be seen in a larger population [34,35]. Future studies examining the effect of diet on FMT outcomes will need to consider the dose and source of dietary fibre. This should include examining differences with and without fibre supplements, specific types of fibre, and the use of objective measures, such as faecal calprotectin in IBDs.

In this pilot study, there was a high degree of variability in the fibre intake of individuals with IBS and IBDs undertaking FMT, with three distinct groups identified. Firstly, those who did not meet the fibre target of 30 g, even with dietary fibre supplementation; secondly, those who exceeded the fibre target, even when excluding dietary fibre supplementation; and thirdly, those who did not meet dietary fibre targets unless including a fibre supplement. The two previous studies that have shown the benefit of fibre supplementation in conjunction with FMT in individuals with UC and constipation utilised the prebiotic supplement pectin at a dose of 20 g and 16 g, respectively. However, these studies did not report on habitual dietary fibre intake [14,15]. Interestingly, a study by Healy et al. has previously demonstrated that participants following a habitually high fibre diet showed a greater gut microbiota response to a prebiotic inulin supplement than those that had a habitually low fibre diet [36]. Overall, the high degree of variability seen in participant fibre intakes highlights diet as a key variable that has not previously been controlled for in FMT intervention studies. This is especially important, as dietary fibre has been frequently demonstrated as a modulator of the gut microbiome [11]. Therefore, standard reporting for future research studies in FMT include habitual diet. Clinically, these considerations suggest that dietary intervention should be customised to patients, which may assist with reducing side effects and increasing the benefits of FMT. Further research is required to determine the benefits of such an intervention, and requires examination of changes in faecal microbiota composition to further elucidate changes. Furthermore, while examination of the diets of FMT donors was outside the scope of this pilot study, this may also be a key variable in improving outcomes after FMT [13]. Thus, future studies examining FMT should include dietary analysis of habitual intake of both recipients and donors.

This study was beneficial in examining the dietary intakes and symptoms of individuals with IBS and IBDs undergoing FMT using validated questionnaires and weighed food records. Limitations of this study are recognized, including the lack of a baseline food diary, lack of stool metagenomics or metabolomics analysis, the small, heterogeneous sample size from a single centre with limited follow-up, and the inherent limitations of weighed food records and self-reported symptoms and QOL measures. The observational nature of the study allowed for associations between diet and FMT outcomes to be explored. However, future controlled trials comparing FMT in conjunction to high fibre diets or supplements to FMT alone are needed to determine causation.

Individuals with IBS and IBDs undertaking FMT do not consistently meet the recommended intakes of fibre, calcium, fruit, vegetables, dairy, or grains. Clinically, greater recognition of self-imposed dietary restrictions is required, and encouragement of the consumption of dairy or calcium-rich alternatives should be considered, especially when individuals are clinically following FMT well. The high degree of variability seen in participant fibre intakes highlights diet (a known modulator of the gut microbiome) as a key variable that has not been previously controlled for in FMT intervention studies. Future studies examining FMT should include dietary analysis of the habitual intake of recipients and donors.

## Figures and Tables

**Table 1:** Baseline demographic data of participants undergoing FMT treatment (*n* = 18).

	Total Cohort	Irritable Bowel Syndrome	Ulcerative Colitis	Crohn’s Disease	*p*-Value *
	(*n* = 18)	(*n* = 7)	(*n* = 4)	(*n* = 7)	
Age *median (range)*	35 (18–57)	34 (25–50)	44 (21–57)	33 (18–55)	0.63
Males *n* (%)	8 (44)	3 (43)	2 (50)	3 (43)	
Bowel motions/day *median (range)*	2 (0–6)	2 (0–3)	1 (1–5)	2 (0–6)	0.73
Abdominal symptoms *n* (%)					
Urgency	10 (55)	3 (43)	2 (50)	5 (71)	
Nausea *n* (%)	10 (55)	5 (71)	2 (50)	3 (43)	
Abdominal pain *n* (%)	9 (50)	4 (57)	2 (50)	3 (43)	
Bloating *n* (%)	14 (78)	6 (86)	3 (75)	3 (43)	
Flatulence *n* (%)	12 (67)	4 (57)	3 (75)	5 (71)	
SF-36 Quality of Life *median (range)*					
Physical functioning	88 (35–100)	80 (35–95)	95 (90–100)	85 (40–100)	0.11
Role physical	25 (0–100)	0 (0–25)	100 (0–100)	75 (0–100)	**0.04**
Bodily pain	58 (0–100)	28 (0–58)	74 (55–90)	78 (33–100)	**0.02**
General health	40 (20–70)	30 (20–60)	65 (40–65)	45 (25–70)	0.05
Vitality	38 (10–75)	30 (10–75)	50 (30–65)	40 (10–70)	0.52
Social functioning	50 (0–100)	19 (0–50)	69 (50–88)	75 (25–100)	**0.01**
Role emotional	33 (0–100)	0 (0–100)	83 (0–100)	67 (0–100)	0.19
Mental health	60 (16–88)	56 (16–68)	68 (52–76)	40 (10–70)	0.33
BMI median (range)	23 (20–29)	22 (20–25)	22 (21–28)	26 (21–29)	0.43

* Kruskal–Wallis H Test to compare IBS, UC, and Crohn’s disease groups. Bolded figures indicate significant.

**Table 2:** Median nutrient intake of participants at week four with and without supplementation (*n* = 14).

Nutrient	EAR/AI	Intake (Including Supplements) Median (Range)	Number of Participants with Intakes (Including Supplements) below the EAR/AI *n* (%)	Intake (Excluding Supplements) Median (Range)	Number of Participants with Intakes (Excluding Supplements) below the EAR/AI *n* (%)
Fibre (g)	30 g	34.9 (15.0–90.5)	6 (43%)	**27.2** (6.0–85.7)	8 (57%)
Vitamin A (µg)	500–625	1105.7 (147.4–4270.6)	1 (7%)	1105.7 (126.6–4270.6)	1 (7%)
Thiamine (B1) (mg)	0.9–1	1.8 (1.1–52.2)	0 (0%)	1.4 (0.5–38.4)	2 (14%)
Riboflavin (B2) (mg)	0.9–1.1	1.8 (1.0–102.8)	0 (0%)	1.5 (0.7–2.7)	1 (7%)
Niacin (B3) (mg)	11–12	24.1 (15.7–521.9)	0 (0%)	21.9 (15.7–58.8)	0 (0%)
Vitamin B6 (mg)	1.1	2.2 (1.4–11.5)	0 (0%)	1.9 (1.0–4.3)	1 (7%)
Folate (B9) (µg)	320	609.1 (405.2–1024.4)	0 (0%)	609.1 (263.7–1024.4)	2 (14%)
Vitamin B12 (µg)	2.0	4.8 (1.1–1000.5)	1 (7%)	4.0 (0.5–5.7)	3 (21%)
Vitamin C (mg)	30	157.5 (45.6–358.1)	0 (0%)	124.7 (14.1–301.5)	1 (7%)
Calcium (mg)	840	**832.1** (356.2–1029.7)	8 (57%)	**786.9** (212.5–998.2)	10 (71%)
Iron (mg)	6–8	14.4 (7.0–35.1)	1 (7%)	12.5 (4.8–28.7)	2 (14%)
Magnesium (mg)	255–350	479.9 (215.8–888.4)	4 (29%)	375.2 (141.4–860.8)	6 (43%)
Zinc (mg)	6.5–12	13.2 (9.3–31.5)	1 (7%)	12.7 (5.1–17.2)	3 (21%)

EAR: Estimated average requirements, AI: adequate intake. Bolded figures indicate median intake below the EAR/AI.

## Data Availability

The data presented in this study are available upon request from the corresponding author. The data are not publicly available due to privacy and ethical reasons.

## Supplementary Materials

The following are available online at https://www.mdpi.com/article/10.3390/nu13051487/s1, Table S1: Pre-treatment antibiotic combinations of patients with Ulcerative Colitis undergoing FMT, Table S2: Energy and macronutrient intake of participants at week 4 (*n* = 14).
