# Supplementary material for: Dietary Intakes of Recipients of Faecal Microbiota Transplantation: An Observational Pilot Study
Source: Nutrients. 2021 Apr 28;13(5):1487. doi: 10.3390/nu13051487 (PMC8147000; doi:10.3390/nu13051487)
Supplement: Supplementary file 1 [file nutrients-13-01487-s001.zip › nutrients-1187953-supplementary.pdf]

Supplementary Materials

Additional Table 1: Pre-treatment antibiotic combinations of patients with Ulcerative Colitis undergoing FMT

**Macronutrient**	**Median (range)**
Energy (kJ)	9268.9 (5966.3-14002.6)
Carbohydrates (g)	194.2 (65.7-386.4)
Protein (g)	105.9 (69.9-245.9)
Fat (g)	91.2 (62.3-138.9)

Additional Table 2: Energy and macronutrient intake of participants at week 4 (n=14)

**Patient ID Number**	**Antibiotic combination**	**Concomitant medications**
107	Rifaximin and Metronidazole	Salofalk
108	Rifampicin and Metronidazole	Purinethol
121	Tinidazole, Nitazoxanide, Rifaximin	Prednisone, Dothep, Salofalk,
126	Nitazoxanide, Tinidazole, Rifaximin	Mezavant
